# The Pathophysiological Role of Heat Shock Response in Autoimmunity: A Literature Review

**DOI:** 10.3390/cells10102626

**Published:** 2021-10-01

**Authors:** Ariadni Androvitsanea, Kostas Stylianou, Eleni Drosataki, Ioannis Petrakis

**Affiliations:** 1 Department of Nephrology, Friedrich Alexander University, 91054 Erlangen, Germany; ariadni.androvitsanea@uk-erlangen.de; 2 Department of Nephrology, University of Crete, 71500 Heraklion, Greece; kstylianu@gmail.com (K.S.); elenidro2@hotmail.com (E.D.)

**Keywords:** heat-shock proteins, autoimmunity, heat-shock response

## Abstract

Within the last two decades, there has been increasing evidence that heat-shock proteins can have a differential influence on the immune system. They can either provoke or ameliorate immune responses. This review focuses on outlining the stimulatory as well as the inhibitory effects of heat-shock proteins 27, 40, 70, 65, 60, and 90 in experimental and clinical autoimmune settings.

## 1. Introduction

Heat-shock proteins (HSPs) are molecular chaperones participating primarily in protein folding preventing protein degradation and subsequent cellular distress [1]. HSPs are regulated through heat-shock factor 1(HSF-1) [2]. In the steady state HSF-1 is bound to HSP90 or HSP70 [3,4]. Upon stressful signals HSF-1 dissociates from HSPs and translocates into the nucleus where it stimulates HSP expression [1]. HSPs can be exposed to the immune system through tissue necrosis and the resultant cellular debris, via organized release of exosomes/endosomes, or through their presence on the cellular membrane [5,6,7]. Their evolutionary conservation can elicit interspecies immune recognition [8]. The resulting immune response can be either immunoregulatory or immunostimulatory [9,10]. Furthermore specific HSP domains as well as certain HSP isoforms and their client proteins induce a differential autoimmune response. The purpose of the present review is to outline the yet known pathophysiology guiding these bimodal and sometimes paradoxical phenomena. The effects of heat-shock protein 27 (HSP27), heat-shock protein 40 (HSP40), heat-shock protein 70 (HSP70), heat-shock protein 60 (HSP60), heat-shock protein 65 (HSP65), and heat-shock protein 90 (HSP90) in eliciting differential immune responses in experimental as well as in clinical autoimmune settings will be described.

## 2. Structural Characteristics, Subcellular Localization of HSPs, and Elicited Immune Responses

### 2.1. Structure and Subcellular Localization of the Small HSP Family

The small HSP (sHSP) gene family has 11 family members (Table S1) [11], which are located in the nucleus, cytoplasm, extracellular space, and the cytoskeleton where they can modulate its structure [12,13,14] 

**Figure 1:**
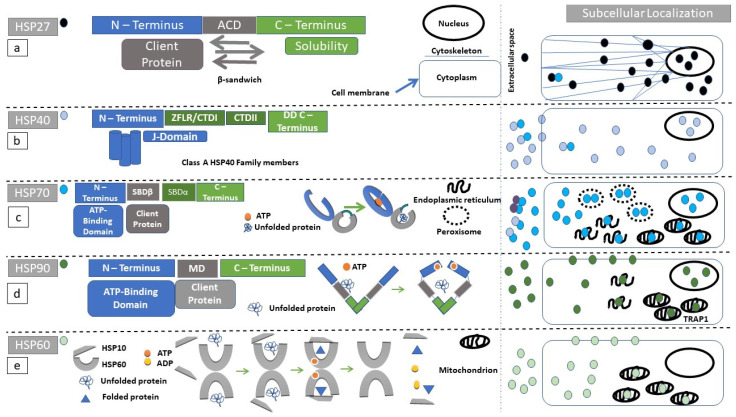
Structure and function of heat shock proteins (HSPs): Diagrammatic representation of the domain structure and subcellular localization of HSPs under discussion. Of note is the fact that heat-shock proteins can form complexes with other molecular chaperones. These chaperone complexes may exert a different action than the uncomplexed HSPs. (**a**) HSP27 (black circle) secondary structure, consists of an N-terminal (blue rectangle) substrate-binding region, followed by an alpha crystallin domain (ACD, gray rectangle) ending in the C-terminus (green rectangle). ACD has a β-sandwich conformation. Client proteins dock to ACD. The C-terminus is highly variable among protein members and facilitates HSP27 oligomerization. (**b**) Class A HSP40 (blue circle) protein family secondary structure consists of an N-terminal (blue rectangle) substrate-binding region, followed by a zinc finger-like region (ZFLR), C-terminal domains I and II (CTDI and II, green rectangles in c-terminal region), and ending in a dimerization domain (DD). The J-domain localizes within N-terminal region. Class B preserves the N-terminal localization of the J-domain but the C-terminus can acquire a more diverse structure. In class C, the J-domain can be localized anywhere within the amino-acid sequence. (**c**) HSP70 (turquoise circle) secondary structure consists of an N-terminal domain (blue rectangle), followed by a substrate-binding domain (SBDβ, gray rectangle), a substrate-binding domain α-helical (SBDα, gray rectangle), and ending in the C-terminus (green rectangle). The reaction cycle involves ATP docking within N-terminal domain since ATP hydrolysis powers the structural opening of the substrate cleft within the SBDβ (gray arc). (**d**) The HSP90 (dark green circle) secondary structure consists of an N-terminus (blue rectangle), followed by a middle domain (MD, gray rectangle), ending in a c-terminus (green rectangle). HSP90 homodimerizes with the use of its c-terminal region. Unfolded proteins are docking in the MD. ATP hydrolysis is required for substrate processing. (**e**) The HSP60 (light green circle) reaction cycle. Unfolded substrates enter the HSP60 processing cleft. HSP10 acts as a lid, and ATP-hydrolysis is necessary for substrate folding.

Small HSPs have a central alpha crystallin domain (ACD) bounded by N-terminal and C-terminal domains (Figure 1a) [15,16,17]. The ACD entails many antiparallel β-sheets which form its final β-sandwich conformation [15]. The N-terminal domain contains serine residues which can be phosphorylated by intracellular kinases. For example, MAPK-activated protein kinase 5 (MK5) can interact with HSP27 in vivo and influence F-actin-dependent cytoskeletal organization [18]. Binding of denatured proteins (client proteins) to sHSPs is characterized by diversity in terms of their docking sites. The N-terminal domain as well as the ACD can serve as client protein-binding sites [15,19]. 

Each one of the sHSPs plays a pivotal role in stabilizing denatured native proteins. They lack, however, the ability to refold destabilized proteins [20], thus sHSP interaction with larger HSPs such as HSP40 or HSP70 is necessary [15]. Larger HSPs, in contrast with sHSPs, have an ATPase function which provides the energy needed to refold the client protein [21]. Normally sHSP molecules are in a polymeric/oligomeric state equilibrium. The presence of noxious stimuli favors their oligomerization. N- and C-termini confer to sHSPs solubility facilitating their oligomerization (Figure 1a) [15,22]. sHSP oligomers can be engaged within protein aggregates in order to facilitate protein folding [23,24]. HSP27 is the most referenced member of the sHSP family in cases of autoimmunity (see below). 

### 2.2. Immune Response Elicited through HSP27

**Figure 2:**
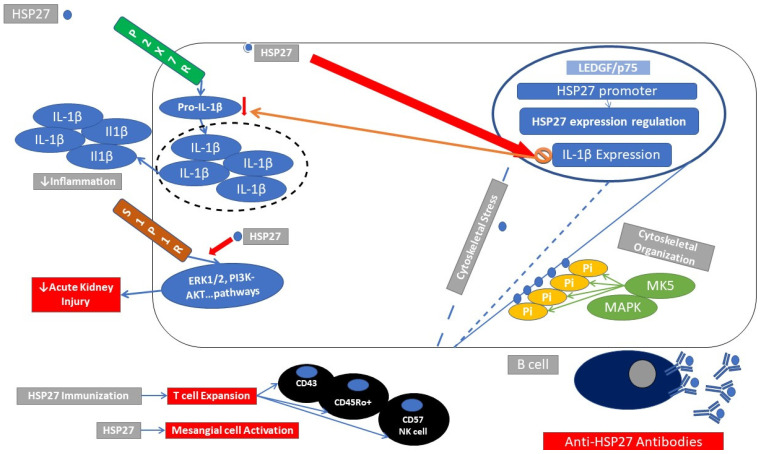
Immunomodulatory actions of HSP27. HSP27 (blue circle) participates in cytoskeletal integrity in cases of cellular distress. Phosphorylation of the N-terminal domain of HSP27 through MAPK kinase protects against cytoskeletal disorganization. HSP27 gene expression can be controlled through transcription factors *LEGF* (lens epithelium growth factor). HSP27 may inhibit mRNA expression of IL-1β and thus inhibit the production of proinflammatory cytokine IL-1β and subsequent inflammatory milieu, P2X7R (ATP-gated P2X cation channel receptor). HSP27 activates SIP1R (sphingocine 1 phosphate receptor) signaling, ameliorates renal inflammation, and protects against acute kidney injury (AKI). Proinflammatory actions of HSP27. HSP27 induces mesangial cell activation; immunization with HSP27 leads to expansion of specific T-cell populations (CD43+, CD45Ro+, and CD57+ NK cells) as well as the production of HSP27 autoantibodies.

The sHSP family, apart from its chaperoning function, has a pivotal role in cytoskeletal organization in conditions of cellular stress, transducing signals after autoantibody stimulation [25] (Figure 2). Aberrant phosphorylation of HSP27 correlates to various clinical pathologies, such as viral infections, specific tumor cells, and autoimmune skin diseases (pemphigus vulgaris and pemphigus foliaceus) [18].

Reduction in HSP27 levels leads to an increase of pro-IL-1β protein in LPS-treated monocytes and HSP27-knockdown cells release significantly more IL-1β [26].

Upregulation of HSP27 was primarily induced by immunoregulatory cytokines such as IL-4, IL-6, and TGF-β, whereas the expression of other sHSPs such as alpha B-crystallin was found solely to be enhanced by the pro-inflammatory cytokine TNFα. Apparently, there is a HSP-specific cytokine combination that provokes or ameliorates its expression [27].

NZBxW/F1 mice develop a spontaneous lupus phenotype manifesting with lupus nephritis. When NZBxW/F1 mice were immunized with recombinant ribosomal protein P0 (rRibos.P), anti-rRibos.P antibodies developed in the context of lupus disease. Primary mesangial cells were exposed to NZBxW/F1-mouse anti-rRibos.P and to human anti-rRibos.P antibodies, respectively. This action induced an activation of mesangial cells partly mediated through HSP27 [28] (Figure 2). 

Myasthenia gravis (MG) is a paraneoplastic syndrome defined by the presence of acetylcholine receptor antibodies (AchR-Abs) which occurs in up to 30% of patients with thymoma. Phosphorylated HSP27 was significantly increased in the serum of patients with MG, who were positive for AchR-Abs compared to seronegative patients [29]. 

In patients with cancer, HSP27 was among the antigens capable of inducing an immunoregulatory action in lymphoid cell lines. In a phase 2 study vaccination of cancer patients with HSP27, client peptides induced lymphoid cell infiltration in the postvaccine biopsy, with an evident increase in the number of total T-cells (CD43+) and mature activated T-cells (CD45Ro+). The postvaccine biopsy also showed an increase in the number of NK-cells (CD57+) [30] (Figure 2). 

Deletion of the endothelial-expressed sphingosine-1-phosphate 1 receptor (S1P1R) is associated with exacerbation of renal injury and cellular inflammatory infiltrates after ischemic acute kidney injury (AKI) in mice. The authors identified an endothelial reduction of HSP27 expression as a mechanism for exacerbated kidney injury and neutrophil infiltration after ischemic AKI in mice (Figure 2). Fingolimod, a S1P1R agonist, is highly protective in ischemic AKI [31,32]. However, fingolimod seems to exert its action through multiple pathways including activation of protein phosphatase 2A (PP2A) and activation of necroptosis [33]. HSP27 externalization has been identified as playing a central role in neutrophilic cell death after fingolimod exposure [33]. This action is mediated through activation of receptor-interacting protein kinase (RIP1/RIP3) and the mixed-lineage kinase domain-like (MLKL) pathway [33].

In the setting of organ transplantation, there is a statistically higher level of serum HSP27 from lung transplant recipients with bronchiolitis obliterans (BOS) compared to control subjects. BOS accompanies chronic lung allograft dysfunction and is characterized by obliterative fibrosis of the small airways [34]. BOS is considered as a manifestation of chronic allograft rejection [34]. Anti-HSP27 antibody levels were significantly higher in broncho-alveolar lavage (BAL) in patients with BOS compared to lung transplant recipients without BOS. Elevated serum levels of HSP27 and elevated antibody titers against HSP27 only in the BAL suggest a localized immune response occurring at the level of alveoli and terminal airways [35].

### 2.3. Structure and Subcellular Localization of HSP40 Family Members

Eukaryotes generally express an expanded arsenal of HSP40s compared to prokaryotes [36]. To date there are 49 human genes coding for separate members of the HSP40 family (Table S2). HSP40 protein family members are localized within the nucleus, plasma membrane, extracellular space, and cytoplasm (Figure 1b) [37,38].

The molecular signature of the HSP40 family is the J-domain, which contains multiple α-helices and has a critical role of stimulating the ATPase domain within HSP70 protein family members [21]. A histidine–proline–aspartate (HPD) motif is required for the J-domain to be functional [21]. HSP40 family members are categorized into type I, type II, or type III, according to their structural conformation. Types I and II have a J-domain located at the N-terminus. In type III, is apparent that the J-domain can be located in any position of the protein sequence [36]. The C-terminal domain of HSP40 binds denatured client proteins [39]. Since both HSP40 and HSP70 family members can be localized in the extracellular space [40,41] they could collectively interact with immune system components.

### 2.4. Immune Response Elicited through HSP40

**Figure 3:**
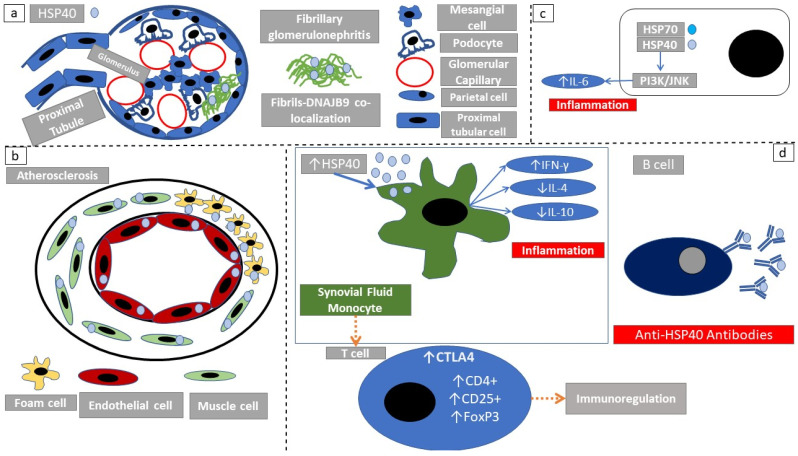
Immunomodulatory actions of HSP40. (**a**) HSP40 is a biomarker of fibrillary glomerulonephritis. The fact that *DNAJB9* colocalizes with fibrils, depicts that HSP40 protein family members have an extracellular function. (**b**) In atherosclerosis, HSP40 is highly expressed in atheromatous lesions. More specifically cellular components that actively participate in atheroma formation express high amounts of HSP40. (**c**) HSP40/HSP70 complexes induce PI3K/JNK signaling and inflammation. (**d**) HSP40 exerts a bimodal action. Antigen-presenting cells exposed to HSP40 induce an inflammatory response through increased IFNγ production. However in a later phase, there is an expansion of tolerogenic T-regulatory cells.

The term glomerulonephritis defines the subset of glomerular diseases in which inflammation or autoimmunity play a substantial pathogenetic role. A member of HSP40 protein family, *DNAJB9*, is a novel biomarker with a sensitivity and specificity near 100% for fibrillary glomerulonephritis [40,42,43]. Fibrillary glomerulonephritis is characterized by the extracellular deposition of non-amyloid fibrils ranging between 16 and 25 nm [42]. Immunoelectron microscopy revealed HSP40 localization to individual fibrils of fibrillary glomerulonephritis [42] (Figure 3a). 

A homolog of the human HSP40, *HDJ-2*, found in *Escherichia coli*, is significantly increased in human atherosclerotic carotid artery plaques when compared with non-atherosclerotic intima (Figure 3b). Furthermore, immunoreactive HDJ-2 protein was localized in macrophage-derived foam cell surfaces, in endothelial cells, and in vascular smooth muscle-like myointimal cells [44]. The authors suggest that HDJ-2 expression may be responsible for T-cell activation in the development of atherosclerosis (Table 1). HSP40 is increased in stroke patients. Increased expression of human HSP40/HSP70 during stroke may lead to autoimmunization against human HSP40 and may cause the immunological cross-reaction against bacterial HSP40 [45]. HSP40 has been shown to stimulate a macrophage cell line (RAW264) to secrete IL-6 through activation of the PI3K and JNK signaling pathways towards a pro-inflammatory response [46,47] (Figure 3c).

HSP40 induces an in vitro decline of the production of the proinflammatory cytokine TNFα and a corresponding increase of the tolerogenic cytokine IL-10 in the synovial fluid of juvenile idiopathic arthritis patients. This decline seems to be dependent on *PD-1* and *CTLA-4* expression [48]. In order to study T- cell responses to HSP40 peptide fragments in patients with oligoarticular juvenile arthritis, Massa et al. [48] showed that proliferative responses of patient synovial fluid monocytes (SFMCs) to recombinant *E. coli* HSP40 (*rdnaJ*) were significantly higher than those of the corresponding peripheral blood monocytes. The exposure of SFMCs to HSP40 peptide fragments induced CD4+, CD25+ high T-cells (Treg) with higher expression of CTLA-4, IL-10, and FoxP3 mRNA. These T-cells had the ability to suppress effector T-cell proliferation in vitro. Although the CD4+, CD25+ high Treg-cells clearly could not prevent the development of the disease, they may contribute to reversing ongoing inflammation. According to this mechanism, patients with persistent oligoarticular juvenile arthritis may have partially maintained the Treg-cell function in response to self-HSP40 in the joint, where it is overexpressed during inflammation; this may result in the self-remitting course of the disease [48] (Figure 3d). HSP40-family-member expression is influenced by external stimuli, more specifically, the presence of the heat-shock proteins *DnaJB4* and *DnaJC6* was higher in the synovial tissue compared to non-smokers with rheumatoid arthritis [49]. These local changes can activate pro-inflammatory signaling pathways and promote autoimmunity.

Bullous pemphigoid is a bullous autoimmune disease of the skin. It is characterized by the presence of auto-antibodies against components of the dermal–epidermal junction. Circulating IgG autoantibodies directed against HSP40 were elevated in patients with active bullous pemphigoid and pemphigus vulgaris compared with healthy controls [50].

The expression of the HSP40 family homolog *DNAJC15* is directly influenced by IFNγ. The reduction of *DNAJC15* expression is regulated through *ikaros*, a transcription-regulating factor, which directly binds the promoter region of *DNAJC15* gene under IFNγ influence. Therefore, the regulation of HSP gene expression involves the participation of proinflammatory cytokines [51].

### 2.5. Structure and Subcellular Localization of HSP70 Superfamily Members

HSP70 family members have a central role in protein unfolding. There are 17 human family members of the HSP70 superfamily (Table S3). HSPA1 is the most studied isoform of HSP70 [8,52]. HSP110 is also a member of HSP70 superfamily [53]. HSP70 localizes in the cytosol, the nucleus [54], the endoplasmic reticulum(ER) [55], the peroxisomes [56], the extracellular space [57,58] and the mitochondria [59] (Figure 1c). Through its extracellular localization, and its complexing with other HSPs, HSP70 may directly present client peptides to the local immunological microenvironment.

What designates the HSP70 superfamily is the N-terminal nucleotide-binding domain (NBD) [21]. NBD has four subdomains (namely IA, IB, IIA, and IIB) surrounding an ATP-binding pocket [60]. C-terminal substrate-binding domain (SBD) has a β-sandwich (SBDβ) and an α-helical domain (SBDα) [60,61]. For ATP-hydrolysis, the binding of J-domain-baring chaperones is necessary (Section 2.3). ATP hydrolysis is a major determinant of its spatial conformation and protein-binding function [5]. By binding ATP, an NBD-binding pocket opens (Figure 1c). Consequently, SBDα is detached from SBDβ and embarks onto NBD [21,61]. As a result of ATP-binding, there is increased affinity and processing rate of non-native peptides [21]. GrpE (GroP-like gene E), BAG (Bcl-2-associated athanogene), proteins with Arm (armadillo repeat) domain, and HSP110 are the nucleotide exchange factors (NEFs) [21,53,62,63,64]. NEFs assure proper substrate release from HSP70 machinery [21,61].

### 2.6. Immune Response Elicited through HSP70

**Figure 4:**
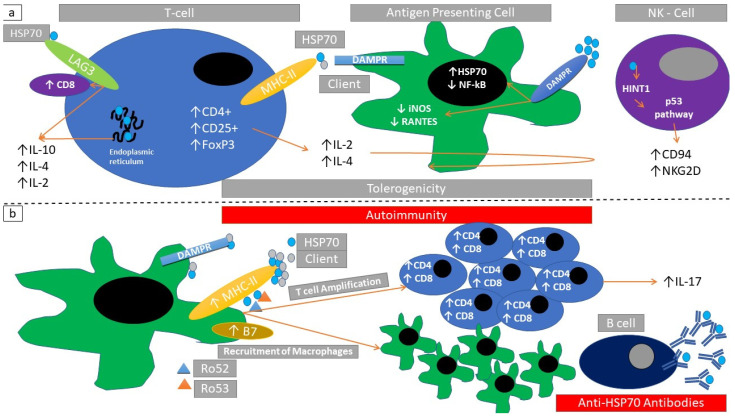
Immunomodulatory actions of HSP70 (**a**) HSP70/client protein-complexes induced signaling through HLA-DR binding in T-regulatory cells. Endoplasmic reticulum HSP70, binding of HSP70 with LAG3 receptor increases IL-2, IL-4, and IL-10. This induces inactivation of antigen-presenting cells (inducible nitric oxide synthase reduction-iNOS, regulated on activation of normal-T-cell-expressed and secreted reduction-RANTES). These changes can also be induced through direct binding of HSP70 with damage-associated molecular pattern receptors (DAMPR). Further exposure to HSP70 can induce *HINT1* (histidine triad nucleotide-binding protein) signal transduction leading to increased expression of *CD94* and *NKG2D* in NK-cells. Collectively these changes promote tolerogenicity. (**b**) HSP70/Ro52 and HSP70/Ro53 complexes induce macrophage infiltration and cytotoxic T-cell infiltration. A parallel autoantibody production against HSP70 may coexist. Collectively these changes promote autoimmunity.

Antigen-presenting cells exposed to HSP70 secrete more TNFα, IL-6, IL-12, and IL-1β, and enhance surface expression of B7 and maturation of immature dendritic cells. HSP70 also binds to its client proteins through the KEFRQ-like motif [65] and leads to MHC-II recognition [6,65,66,67,68] (Figure 4b and Table 2). An HSP70-associated expansion of T-cells was observed. This T-cell expansion was CD4-dependent but not CD28-dependent [68]. Millar et al. showed that the immunization of RIP-GP/P14 mice with recombinant HSP70 (rhHSP70) induced the onset of diabetes showing an in vivo promotion of autoimmunity [68]. 

*HSPA5* (an endoplasmic reticulum isoform of HSP70) elicits an immunomodulatory T-cell response (increase of IL-10 and IL-4 production) diminishing experimental autoimmune arthritis activity [69]. Multiple HSP70 client peptides promote a T-regulatory cell phenotype(CD4+, CD25+, FoxP3+) [70]. T-regulatory cell stimulation through HSP70, induced an increase in *LAG3* expression (CD233 induces the suppressive function of T-regulatory cells [70,71]) (Figure 4a). In a mouse model of autoimmune arthritis, T-regulatory cell expansion and the subsequent suppression of disease activity was mediated through the *LAG3* co-stimulatory molecule [70]. 

Furthermore, HSP70 is present in clathrin-coated pits, uncoating during clathrin-mediated endocytosis. HSP70 packaged in exosomes can be released from cells. This attracts T-cells bearing a CD8+ IL-10+ phenotype [65]. 

HSP70 and client peptide *HINT1* (histidine triad nucleotide-binding protein-1, a protein having an active role in the p53 signaling pathway [52]) mediate immunoregulation through CD94 and *NKG2D* (NKG2-D type-II integral membrane protein; a costimulatory receptor of NK-cells [72]) signaling in a mouse model of experimental autoimmune encephalomyelitis (EAE) [73]. Detection of HSP70 mRNA was related with reduced clinical inflammation scores in an experimental mouse model of EAE. In this set of experiments, there was a reduction in inducible nitric oxide synthase (NOS) production, *RANTES* (chemokine C-C motif ligand 5), and *NF-κB* mRNA [74] (Figure 4a). This shows that HSPs can regulate gene expression in response to autoimmune stimuli. 

Immunization of Balb-c mice against α-actinin induces autoimmune responses against HSP70 and produces a lupus-like phenotype [75]. HSP70 dermis exposure causes inflammatory infiltration and increases IL-6 production with progressive reactivity of T-cytotoxic cell phenotypes (CD4+, CD8+) through IL-17 production [76] (Figure 4b).

Ro52 and Ro60 complex with Grp78 (an inducible form of HSP70). These complexes are recognized via surface immunoglobulins specific for the HSP70 component [77]. Grp78/Ro52 complexes co-localize with HSP90 in apoptotic debris and stimulate T-cells [78].

In salt-sensitive hypertension there is an overexpression of tubulointerstitial HSP70, T-cell proliferation with perivascular T-cell infiltration and circulating anti-HSP70 antibodies [79]. HSP70 was found increased in a cohort of ANCA (anti-neutrophil cytoplasmic antibody) vasculitis patients. Increased presence of interstitial HSP70 was associated with worsened kidney survival in this cohort [80]. There is a plethora of examples showing that elevated serum circulating anti-HSP70 correlates with immune response modulation in humans as well as in laboratory animals (Table 1 and Table 2).

### 2.7. Structure and Subcellular Localization of HSP90

There are five members of the human HSP90 family. The HSP90 family members (Table S4) are localized within the cytoplasm, the endoplasmic reticulum, the endosomes [41], the cell membrane [81], and the nucleus [82], and they can be secreted in the extracellular space (Figure 1d) [41]. Furthermore, the HSP90 isoform, *TRAP1*, is localized within the mitochondrion [83]. HSP90 interacts with other client proteins as well as other members of the heat-shock-protein family [84]. The N-terminal domain of HSP90 contains its ATP-binding pocket [85]. This N-terminal domain is followed by a middle domain leading to the C-terminal region [86] (Figure 1d). The middle domain is responsible for client protein-binding [86]. The C-terminal region of HSP90 homodimerizes in the steady state. By binding of ATP in its N-terminal domain, HSP90 gains its active conformation [87]. After completion of its chaperone function, the ATP molecule is hydrolyzed and HSP90 regains its resting state [86]. 

### 2.8. Immune Responses Elicited through HSP90

**Figure 5:**
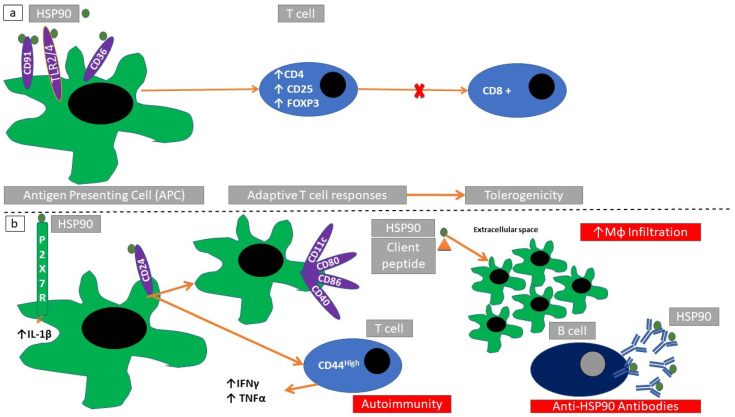
Immunomodulatory actions of HSP90. (**a**) Through binding with specific receptors (TLR2/9, CD36, and CD91) on antigen-presenting cells, HSP90 blocks cytotoxic T-cell expansion (CD8+) and induces adaptive T-cell responses. (**b**) Through binding with specific receptors (ATP-gated P2X cation channel receptor, P2X7R) HSP90 induces the production of proinflammatory cytokines (IL-1β). Binding to CD25 can induce macrophage activation and further propagate immunoreactive T-cell responses. This can be followed by macrophage recruitment and anti-HSP90 antibody production.

Exposure to an HSP90 isoform (*grp96*) downregulates T-cell responses in experimental models of type 1 diabetes mellitus and EAE. HSP90 causes an expansion of CD4+ T-regulatory cells by binding to CD91, CD36, and TLR2/4, which in turn can inhibit CD8+ T-cells [88]. Exposure of dendritic cells to grp96 suppresses their maturation. PGMA1 (2,3-bisphosphoglycerate-dependent phosphoglycerate mutase, an enzyme participating in glycolysis pathway) complexes with grp96 producing an immunosuppressive effect [89]. Inhibition of HSP90β with vibsanin-B inhibits interstitial leukocyte migration in a mouse model of EAE [90]. Small inhibiting-RNA (si-RNA)-induced inhibition of grp96 prevents dendritic cell maturation without involving TLR4 signaling. AIMP1 (aminoacyl tRNA synthase complex-interacting multifunctional protein 1, a multirole protein involved in many disease processes including immune modulation [91]) binds grp96 and reduces the intensity of the elicited immune response [92] (Figure 5a). Functional HSP90 is required for P2X7-receptor-mediated IL-1β release in a mouse model of autoimmune exocrinopathy [93] (Figure 5b). Exposure of HSP90 on the cell membrane is associated with a lupus-like phenotype, mediated through CD24+ antigen-presenting cells. Extrapolating data from a CD24-knockout mouse model, HSP90 induction of autoimmunity is mediated through regulation of CD11c+ macrophages and inactivation of a specific dendritic cell subset (CD80+, CD86+, CD40+, IL-12+) [7] (Figure 5b).

In a mouse model of collagen type VII autoimmune disease, blocking of HSP90 reduces anti-collagen type VII antibodies, reduces expansion of CD3+, CD28+ T-cells, and increases neutrophil infiltration [94]. Expression of the HSP90 isoform (gp96) on the cell surface is associated with glomerulonephritis and auto-antibody production (anti-nuclear antibodies and anti-dsDNA antibodies). This phenotype is associated with CD4-T-cell stimulation which in turn activates dendritic cells [95].

It is apparent that an armamentarium of different receptors and signaling pathways may have different immune effects upon the exposure to the same HSP. This fact may lead to immunoregulation or immunostimulation.

### 2.9. Structure and Subcellular Localization of Chaperonins

There are 15 members in the human chaperonin family (Table S5). HSP60 and HSP10 are primarily located within the mitochondrion [96], although cell membrane [97,98,99], peroxisomes [96] and extracellular localizations [100] have been reported. The protein structure of this family consists of two heptameric ring subunits, or two octameric rings in the case of TCP1 [101], which come positioned “back to back” to form a barrel-like structure (Figure 1e). Unfolded proteins bind to each subunit ring with 1:1 stoichiometry [102]. HSP10 forms a heptameric cover upon the substrate cavity of HSP60 [103] and is a necessary component of the optimal processing of substrates through HSP60 machinery [104]. ATP-binding, followed by binding of HSP10 can induce processing of the unfolded protein within the central cavity of each ring subunit [102]. As a result, ATP hydrolysis induces dissociation of HSP10, ADP and the release of the folded substrate protein [102].

The molecular chaperone HSP65 is mainly expressed in the cytoplasm of non-mammalian cells, such as *Mycobacterium* species. HSP65 has a very similar structure and function to that of HSP60 and can form oligomeric aggregates within the cell as well as within the extracellular space [105]. The homology between human HSP60 and HSP65 makes molecular mimicry unavoidable, leading to an involvement in immune processes.

### 2.10. Immune Responses Elicited through HSP60

T-cell subpopulations and related responses are classified according to cytokine expression profiles and surface expression molecules. More specifically, T-helper 1 (Th1) cells produce among others, IFNγ, GM-CSF (granulocyte macrophage colony-stimulating factor) and TNFα and promote a proinflammatory state. Whereas T-helper 2 (Th2) cells produce among others IL-4, IL-5, and IL-13 and promote a tolerogenic immune phenotype [106].

**Figure 6:**
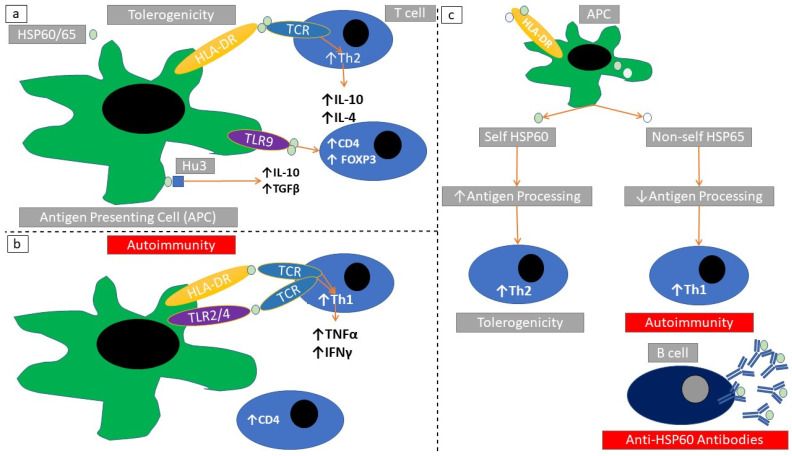
Immunomodulatory actions of HSP60/65. (**a**) HSP60/65 induce a Th2 cytokine response after stimulating TLR9/HLA-DR in antigen-presenting cells. (**b**) HSP60/65 induce a Th1 cytokine response after stimulating TLR2/HLA-DR in antigen-presenting cells. (**c**) Self HSP60 undergoes a complete antigen processing within antigen-presenting cells. This induces Th2 responses and tolerogenicity. Non-mammalian HSP65 undergoes an incomplete antigen processing within antigen-presenting cells. This induces Th1 responses and autoimmunity. Non-self and self HSPs share a conserved amino acid sequence homology. Self-HSP60 can stochastically activate Th1-cell clones. This induces autoimmunity after stimulation with non-self HSP65 molecules. In the case of autoimmunity there can be a parallel production of anti-HSP60/65 autoantibodies.

Molecular mimicry between endogenous and foreign peptides could induce autoimmune phenomena [107]. Mycobacterial HSP65 undergoes a self-induced autolysis engaging MHC-I and MHC-II antigen processing [108]. HSP60/65 are HLA-DR binders and could thus ease client peptide presentation to antigen-presenting cells. This specific binding properties of distinct HSP60/65 peptide regions induces TNFα and IFNγ production [109] (Figure 6a,b). There seems to be a differential T-cell reaction against indigenous compared with exogenous HSP60. While mycobacterial HSP60 induces T-cell activation, the indigenous HSP60 induces T-cell anergy [110] (Figure 6c). 

Type 1 diabetes mellitus is an autoimmune disease [111]. In an experimental model of streptozotocin-induced diabetes, HSP60 inhibited diabetes progression by eliciting a Th2 response [112]. Non-obese diabetic (NOD) mice are a primary animal model for studying autoimmune diabetes [113]. Immunization of NOD mice with a HSP60-p277 peptide, also induces a Th2 response with the subsequent production of IL-4 and IL-10. This is accompanied by reduced immune reactivity against HSP60 through Th1 response downregulation [114]. Immunization with mycobacterial HSP65 has been shown to prevent autoimmune diabetes in NOD mice [115]. On the other hand, HSP60 induced T-cell stimulation which was associated with diabetes aggravation and anti-HSP60 antibody production in experimental models of type 1 diabetes mellitus [116].

Vascular-associated lymphoid tissue (macrophages, T-cells, and mast cells) is stimulated by HSP60 exposure. This stimulation could aggravate atherosclerosis [117]. Autoantibodies against HSP60 were detected after chlamydial infection in cholesterol-fed C57Bl/6 mice and subendothelial accumulation of foam cells was observed [118].

Autoantibodies against HSPs indicate the involvement of humoral immunity in the response induced by HSPs (Table 1). However, it seems apparent that specific subsets of B-lymphocytes are involved [119]. Anti-HSP60 protein antibodies are present in patients with rheumatoid arthritis, SLE, Sjögren syndrome, and undifferentiated connective tissue disease [120,121,122] (Table 1).

In a rat arthritis model, HSP60 induces a TLR9-mediated T-regulatory cell (CD4+, FoxP3+) proliferation leading to IL-10 production. Rats treated with a HSP60 showed greater amount of T-regulatory cells in the joint-draining lymph nodes and had lower arthritis activity scores [123] (Table 2). There are specific domains of HSP65, which exert their immunomodulatory action in autoimmune arthritis; for example, HSP65 peptide P118-388 causes T-cell expansion while HSP65 peptide P180-188 does not inhibit autoimmune arthritis [124]. Bacterial HSP65 protects against arthritis by inducting tolerogenic T-cell clones against self HSP60 [125]. C-terminal mycobacterial HSP65 causes cross reactivity against rat HSP65 in experimental autoimmune arthritis [126].

The conserved sequences of self and non-self HSP60/65 seem to activate the immune system. Unanswered remains the fact concerning the exact amount of non-self HSPs that could induce a cross-recognition reaction from the native immune system. It may be the case that this could be organism-, disease-, tissue-, or target-HSP (or HSPs)-specific. 

## 3. Therapeutic Implications

As of today, there are at least 54 studies concerning therapeutic applications of heat-shock proteins (source: https://clinicaltrials.gov/, accessed on 18 August 2021). The vast majority of those completed, did not concern autoimmune disorders per se. Of those actively recruiting or ongoing, none concern autoimmune disorders. This observation denotes not only the necessity for establishing new treatment strategies but also the complexity of the heat-shock-protein system itself. A multimodal approach which targets multiple heat-shock proteins and components of the immune system may be necessary. Given the fact that the heat-shock-protein system produces an effect in multiple levels of the immune system, the effects of a heat-shock-protein driven intervention might be time demanding. In the aforementioned paradigms, HSPs undergo control at the level of gene expression both by components of the immune system and also by external stimuli. Possible gene polymorphisms of heat-shock response genes might help us individualize future treatments. Given the fact that heat-shock proteins finely modulate immune responses one may have to examine the combined effect of heat-shock response and other immunomodulatory agents. 

At last, but not least, heat-shock proteins can help us drive therapy in various autoimmune diseases. There is relative new knowledge that heat-shock proteins are unique biomarkers of disease (the paradigm of fibrillary glomerulonephritis and the HSP40 isoform *DNAJB9*). Taking this idea one step further, one could use the tissue signature of HSP-system components for guiding therapy in various autoimmune diseases. Utilizing proteomic analysis or immunohistochemistry, HSPs that are uniquely expressed at the tissue level in specific disease stages can guide the intensity as well as the modalities of immunosuppressive therapy.

## 4. Conclusions

It is the very nature of the immune response that is characterized by plasticity. This plasticity is depicted in the case of HSPs: 

(1) Through molecular mimicry in cases of microbial or mycobacterial infection. Infection causes exposure of non-self-antigens to the immune system. The evolutionary conservation of heat-shock proteins induces cross-reactivity with self HSP antigens. 

(2) Through induction of different signaling pathways via a plethora of membrane receptors and client peptides. HSPs act as vehicles which present self-antigens to immune cells. Specific domains within the HSP molecule are responsible for effective antigen presentation. The degree of antigen processing within antigen-presenting cells guides not only the intensity of immune response but also whether this response leads to autoimmunity or immunoregulation. Making things even more complicated, different receptors (MHC-II, TLR, etc.), upon stimulation with different HSPs, produce different immune responses. It seems therefore that the HSP system is dependent on external stimuli and the tissue microenvironment. Immune responses can be finely tuned through exposure to HSPs. Altogether, the above points pose an intriguing endeavor in understanding immunity and planning future therapeutic strategies for autoimmune diseases.

## Figures and Tables

**Table 1:** Heat-shock proteins (HSP) in human autoimmune disease.

Heat-Shock Protein (HSP)	Disease	Effect	References
HSP27	Glaucoma—increased intraocular pressure	HSP27 serum auto-antibodies	[127]
	Myasthenia gravis	Increased HSP27 phosphorylation	[18]
	T-cell neoplasia (thymoma, T-cell carcinoma)	Increased serum HSP27 protein, increased HSP27 tissue expression, patient subsets with reduced expression associated with worsened outcome	[45]
	Lung transplantation	Bronchioalveolar lavage HSP27 auto-antibodies associate with bronchiolitis obliterans	[49]
	Immunization of cancer patients (renal-, breast-, colon-carcinoma, melanoma, and astrocytoma)	Increased immunoreactivity following HSP27 vaccination	[128]
	Guillain Barret	HSP27 serum auto-antibodies	[129]
HSP40	Fibrillary glomerulonephritis	Colocalization of HSP40 with fibrils	[65,69]
	Bullous pemphigoid, pemphigus vulgaris	HSP40 serum auto-antibodies	[66]
	Cigarette smoking and rheumatoid arthritis	HSP40 serum auto-antibodies, HSP40 increase in synovial fluid and worsened clinical course	[44]
	Stroke	HSP40 serum auto-antibodies	[50]
	Various arthritis phenotypes	Complex immunoregulatory or immunostimulatory action	[67]
	Atherosclerosis	Increased HSP40 in atheromatous lesions—implication in pathogenesis	[70]
HSP70	Thyroiditis	HSP70 serum auto-antibodies	[99]
	Inner ear disease	HSP70 serum auto-antibodies, HSP70 associates with steroid responsiveness	[112,114]
	Diabetic microangiopathy	Association of HSP70 serum autoantibodies and disease severity	[110]
HSP90	SLE	HSP90 autoantibodies, HSP90 presence in peripheral blood monocytes	[130]
	HCV infection	Interaction of HSP90 with HCV antigens	[131]
HSP60/65	Systemic lupus erythematosus(SLE), Sjögren syndrome, undifferentiated connective tissue disease, Bechcet’s disease, relapsing polychondritis	HSP60/65 auto-antibodies	[132,133,134]
	Rheumatoid arthritis	HSP60/65 auto-antibodies, modification of immune response, T-cell expansion	[128,129,130]
	Coronary artery disease	Molecular mimicry, worsening of disease activity, presence of autoantibodies	[105,115,135,136]
	Heart transplantation	Worst prognosis co-related with serum autoantibodies	[125]
	*Helicobacter pylori* infection	Presence of autoantibodies	[87]
	Autoimmune hepatitis, hepatitis C virus (HCV) infection	Presence of autoantibodies, interaction with client proteins	[84]
	Renal transplantation	Increased renal HSP65 protein expression associated with Th2 cell shift.	[9]

**Table 2:** Autoimmune effects of heat-shock proteins in animal models.

Heat-Shock Protein (HSP)	Disease Model	Effect	Reference
HSP27	NZBW mice—systemic lupus erythematosus	Lupus nephritis, mesangial cell activation	[30]
	Rat model of glaucoma (increased intraocular pressure, IOP)	HSP27 auto-antibodies in cerebrospinal fluid	[127]
HSP40	Rheumatoid arthritis mouse model	HSP40 auto-antibodies, increased disease activity	[44]
HSP70	Autoimmune arthritis mouse model	Suppression of T cells	[74]
	Mouse model of experimental autoimmune encephalomyelitis(EAE)	Natural-killer-cell-induced immunoregulation, increased HSP70 mRNA associated with reduced inflammation, HSP70 induces a Th17 cell response	[79,137,138]
	Mouse model of salt-sensitive hypertension	Increased renal inflammatory infiltration	[139]
HSP90	Mouse model of type I diabetes mellitus	Immunization with HSP90 reduces autoimmunity	[88,90]
	Mouse model of EAE	Reduction of autoimmune response	[90]
	Mouse models of bullous pemphigoid and pemphigus vulgaris	Reduction of autoimmune response	[140]
	Mouse model of autoimmune exocrinopathy	Increased autoimmunity	[93]
	Mouse model of anti-collagen VII autoimmunity	Increased infiltration of inflammatory cells	[94]
	Rat model of autoimmune arthritis	Immunization reduced arthritis activity, tolerogenicity induction	[124,141]
	Mouse model of hemolytic anemia	Immunization with HSP60/65 reduced autoantibodies against erythrocytes.	[124]
	Rat model of uveitis	Increased activity of uveitis	[122]
HSP60/65	Mouse model of type I diabetes (DM)	Immunization vs HSP60/65 reduced DM severity, immunization increased DM severity and autoimmune response	[116,118,142]
	Mouse model of autoimmune arthritis	Immunization against HSP60/65 reduced arthritis activity, immunization against mycobacterial HSP65 increases arthritis severity	[107,108,143]
	Mouse model of atherosclerosis	Immunization against HSP60/65 increased inflammatory response in atheromatous vascular lesions	[144]
	Mouse model of intestinal autoimmune disease	Increase of intestinal autoimmune lesions	[121]
	Rat model of autoimmune arthritis	Immunization reduced arthritis activity, tolerogenicity induction	[124,141]
	Mouse model of hemolytic anemia	Immunization with HSP60/65 reduced autoantibodies against erythrocytes.	[124]
	Rat model of uveitis	Increased activity of uveitis	[122]

## Supplementary Materials

The following are available online at https://www.mdpi.com/article/10.3390/cells10102626/s1, Table S1: Nomenclature of human small HSP family gene members (modified according to Kampinga et al. [11] and HUGO Gene Nomenclature Committee), Table S2: Nomenclature of human HSP40 family members (modified according to Kampinga et al. [11] and HUGO Gene Nomenclature Committee), Table S3: Nomenclature of human HSP70 superfamily members including HSP70 and HSP110 families (modified according to Kampinga et al. [11] and HUGO Gene Nomenclature Committee), Table S4: Nomenclature of HSP90 family members (modified according to Kampinga et al. [11] and HUGO Gene Nomenclature Committee), Table S5: Nomenclature of chaperonin family members (modified according to Kampinga et al. [11] and HUGO Gene Nomenclature Committee).
