# Supplementary material for: The Pathophysiological Role of Heat Shock Response in Autoimmunity: A Literature Review
Source: Cells. 2021 Oct 1;10(10):2626. doi: 10.3390/cells10102626 (PMC8533860; doi:10.3390/cells10102626)
Supplement: Supplementary file 1 [file cells-10-02626-s001.zip › cells-1349684-supplementary.pdf]

cells MDPI

Table S1. Nomenclature of human small HSP family gene members (Modified according to Kampinga et al[1] and HUGO Gene Nomenclature Committee). Citations elucidate the biological role of each protein component.

Heat Shock Protein	Full Name	Aliases
**HSPB1**	Heat Shock Protein family B(small) member 1	HSP27, HSP28, hsp25, CMT2F (Charcot-Marie-Tooth disease, Type 2F)[2]
**HSPB2**	Heat Shock Protein family B(small) member 2	MKBP(Myotonic Dystrophy Kinase Binding Protein)[3], Hs.78846
**HSPB3**	Heat Shock Protein family B(small) member 3	HSPL27 (27 KDa heat-shock-protein-like)[4]
**CRYAA**	Crystallin alpha A	CRYA1, HSPB4
**CRYAB**	Crystallin alpha B	CRYA2, HSPB5
**HSPB6**	Heat Shock Protein family B(small) member 6	Hsp20[5], FLJ32389, PPP1R91
**HSPB7**	Heat Shock Protein family B(small) member 7	cvHSP (cardiovascular HSP)
**HSPB8**	Heat Shock Protein family B(small) member 8	H11, E2IG1(Estradiol Induced Gene 1)[6], HSP22, CMT2L(Charcot-Marie-Tooth disease, Type 2L)[7]
**HSPB9**	Heat Shock Protein family B(small) member 9	CT51
**ODF1**	Outer dense fiber of sperm tails 1	ODFPG, ODF27, RT7, HSPB10, CT133
**HSPB11**	Heat Shock Protein family B(small) member 10	HSPCO34, PP25, IFT25 (Intraflagellar transport protein 25)[8], FAP232

Table S2. Nomenclature of human HSP40 family members (Modified according to Kampinga et al[1] and HUGO Gene Nomenclature Committee). Citations elucidate the biological role of each family member.

Heat Shock Protein	Full Name	Aliases
**DNAJA1**	DnaJ heat shock protein family (Hsp40) member A1	HSPF4, hdj-2 (human DnaJ homologue 2)[9], dj-2, NEDD7 (Neural precursor cell expressed, developmentally down-regulated 7)
**DNAJA2**	DnaJ heat shock protein family (Hsp40) member A2	HIRIP4 (HIRA Interacting protein 4), DNAJ, CPR3 (Cell cycle progression restoration gene 3 protein)[10], DNJ3
**DNAJA3**	DnaJ heat shock protein family (Hsp40) member A3	hTid-1 (human tumorous imaginal discs homolog)[11]
**DNAJA4**	DnaJ heat shock protein family (Hsp40) member A4	PRO1472
**DNAJB1**	DnaJ heat shock protein family (Hsp40) member B1	**Hsp40,** Sis1, RSPH16B (Radial spoke 16 homolog B)
**DNAJB2**	DnaJ heat shock protein family (Hsp40) member B2	HSPF3
**DNAJB3**	DnaJ heat shock protein family (Hsp40) member B3	HCG3
**DNAJB4**	DnaJ heat shock protein family (Hsp40) member B4	HLJ1 (Human Liver Dna J like protein)[12]
**DNAJB5**	DnaJ heat shock protein family (Hsp40)	Hsc40 (Heat shock cognate 40)[13]

	member B5	
**DNAJB6**	DnaJ heat shock protein family (Hsp40) member B6	MRJ
**DNAJB7**	DnaJ heat shock protein family (Hsp40) member B7	HSC3
**DNAJB8**	DnaJ heat shock protein family (Hsp40) member B8	MGC33884, CT156
**DNAJB9**	DnaJ heat shock protein family (Hsp40) member B9	
**DNAJB11[14]**	DnaJ heat shock protein family (Hsp40) member B11	EDJ, HEDJ (Human DnaJ Protein 9), ERdj3 (Endoplasmic reticulum DNA J domain containing protein 3)
**DNAJB12**	DnaJ heat shock protein family (Hsp40) member B12	DJ10, FLJ20027
**DNAJB13**	DnaJ heat shock protein family (Hsp40) member B13	TSARG6 (Testis spermatocyte apoptosis-related gene 6), RSPH16A (Radial spoke 16 homolog A)[15]
**DNAJB14**	DnaJ heat shock protein family (Hsp40) member B14	FLJ14281[16]
**DNAJC1**	DnaJ heat shock protein family (Hsp40) member C1	DNAJL1 (DNA J like protein 1), ERdj1, MTJ1[17]
**DNAJC2**	DnaJ heat shock protein family (Hsp40) member C2	MPP11 (M phase phosphoprotein 11)[18], MPHOSPH11, ZUO1 (zuotin related factor 1), zuotin
**DNAJC3**	DnaJ heat shock protein family (Hsp40) member C3	P58, P58IPK (Protein kinase inhibitor of 58 KDa)[19], HP58, ERdj6, p58(IPK)
**DNAJC4**	DnaJ heat shock protein family (Hsp40) member C4	MCG18(multiple endocrine neoplasia type 1 candidate gene 18) [20]
**DNAJC5**	DnaJ heat shock protein family (Hsp40) member C5	FLJ00118[21], FLJ13070, DNAJC5A
**DNAJC5B**	DnaJ heat shock protein family (Hsp40) member C5 beta	MGC26226, CSP-beta (Cysteine string protein beta)[22]
**DNAJC5G**	DnaJ heat shock protein family (Hsp40) member C5 gamma	FLJ40417, CSP-gamma(Cysteine string protein gamma)
**DNAJC6**	DnaJ heat shock protein family (Hsp40) member C6	KIAA0473, PARK19(Putative tyrosine-protein phosphatase auxillin)[23,24]
**DNAJC7**	DnaJ heat shock protein family (Hsp40) member C7	TPR2(Tetratricopeptide repeat protein 2)[25]
**DNAJC8**	DnaJ heat shock protein family (Hsp40) member C8	SPF31(Splicing protein spf31)[26]
**DNAJC9[27]**	DnaJ heat shock protein family (Hsp40) member C9	JDD1(J domain of DnaJ-like-protein 1)[28], SB73
**DNAJC10**	DnaJ heat shock protein family (Hsp40) member C10	ERdj5[29], PDIA19(Protein disulfide isomerase family A, member 19)

**DNAJC11**	DnaJ heat shock protein family (Hsp40) member C11	FLJ10737[30]
**DNAJC12**	DnaJ heat shock protein family (Hsp40) member C12	JDP1(J domain containing protein 1)[31]
**DNAJC13**	DnaJ heat shock protein family (Hsp40) member C13	RME8 (required for receptor mediated endocytosis 8)[31], KIAA0678
**DNAJC14**	DnaJ heat shock protein family (Hsp40) member C14	DNAJ, DRIP78(Dopamine receptor-interacting protein of 78 kDa)[32], HDJ3, LIP6 (LYST-interacting protein 13), FLJ32792
**DNAJC15**	DnaJ heat shock protein family (Hsp40) member C15	MCJ(Methylation controlled J protein)[33]
**DNAJC16**	DnaJ heat shock protein family (Hsp40) member C16	KIAA0962[34]
**DNAJC17**	DnaJ heat shock protein family (Hsp40) member C17	FLJ10634[35]
**DNAJC18**	DnaJ heat shock protein family (Hsp40) member C18	MGC29463[36]
**DNAJC19**	DnaJ heat shock protein family (Hsp40) member C19	TIMM14(Mitochondrial import inner membrane translocase subunit TIM14)[37], Tim14, Pam18
**HSCB**	HscB mitochondrial iron-sulfur cluster cochaperone	HSC20[38], DNAJC20, Jac1
**DNAJC21[39]**	DnaJ heat shock protein family (Hsp40) member C21	GS3, DNAJA5, JJJ1
**DNAJC22**	DnaJ heat shock protein family (Hsp40) member C22	Wus (wurst homolog)[40], FLJ13236
**SEC63**	SEC63 homolog, protein translocation regulator	SEC63L, PRO2507, ERdj2, DNAJC23
**DNAJC24**	DnaJ heat shock protein family (Hsp40) member C24	JJJ3
**DNAJC25**	DnaJ heat shock protein family (Hsp40) member C25	bA16L21.2.1
**DNAJC26**	cyclin G associated kinase	GAK(Cyclin – G – associated kinase)[41]
**DNAJC27**	DnaJ heat shock protein family (Hsp40) member C27	RabJS (Ras-associated protein Rap1)[42]
**DNAJC28**	DnaJ heat shock protein family (Hsp40) member C28	C21orf78
**SACS**	sacsin molecular chaperone	ARSACS, KIAA0730, DKFZp686B15167, DNAJC29, SPAX6[43], PPP1R138(Protein phosphatase 1, regulatory subunit 138)
**DNAJC30[44]**	DnaJ heat shock protein family (Hsp40) member C30	

Table S3. Nomenclature of human HSP70 superfamily members including HSP70 and HSP110 families (Modified according to Kampinga et al[1] and HUGO Gene Nomenclature Committee). Citations elucidate the biological role of each family member.

Heat Shock Protein	Full Name	Aliases
**HSPA1A**	heat shock protein family A (Hsp70) member 1A	HSP70-1[45]
**HSPA1B**	heat shock protein family A (Hsp70) member 1B	HSP70-2[45]
**HSPA1L**	heat shock protein family A (Hsp70) member 1 like	HSP70-HOM[46], hum70t
**HSPA2[47]**	heat shock protein family A (Hsp70) member 2	
**HSPA4**	heat shock protein family A (Hsp70) member 4	HS24/P52[48], HSPH2
**HSPA4L**	heat shock protein family A (Hsp70) member 4 like	APG-1[49], Osp94 (Osmotic stress protein 94), HSPH3
**HSPA5**	heat shock protein family A (Hsp70) member 5	BiP (Binding immunoglobulin protein)[50]
**HSPA6**	heat shock protein family A (Hsp70) member 6	HSP70B´[51]
**HSPA7**	heat shock protein family A (Hsp70) member 7	HSP70B[52]
**HSPA8**	heat shock protein family A (Hsp70) member 8	HSC71 (heat shock cognate 71 protein)[53], HSC70, HSP73
**HSPA9**	heat shock protein family A (Hsp70) member 9	GRP75(glucose regulated protein 75)[54], PBP74 (Peptide Binding Protein 74), mot-2(mortalin-2), mthsp75
**HSPA12A[55]**	heat shock protein family A (Hsp70) member 12A	FLJ13874, KIAA0417
**HSPA12B[55]**	heat shock protein family A (Hsp70) member 12B	dJ1009E24.2
**HSPA13[56]**	heat shock protein family A (Hsp70) member 13	
**HSPA14**	heat shock protein family A (Hsp70) member 14	HSP70-4, HSP70L1(HSP70 like protein 1)[57]
**HSPAH1**	heat shock protein family H (Hsp110) member 1	HSP105B[58], KIAA0201, HSP105A, NY-CO-25 (Antigen NY-CO-25)
**HYOU1**	Hypoxia up-regulated 1	ORP150(oxygen regulated protein 150)[59], HSP12A, Grp170 (Glucose regulated protein 170)[60]

Table S4. : Nomenclature of HSP90 family members (Modified according to Kampinga et al[1] and HUGO Gene Nomenclature Committee). Citations elucidate the biological role of each family member.

Heat Shock Protein	Full Name	Aliases
**HSP90AA1**	heat shock protein 90 alpha family class A member 1	Hsp89, Hsp90,

		FLJ31884,<br>HSP90N
**HSP90AA3P**	heat shock protein 90 alpha family class A member 1, pseudogene	
**HSP90AB1[61]**	heat shock protein 90 alpha family class B member 1	
**HSP90B1**	heat shock protein 90 beta family member 1	GP96[62],<br>GRP94(glucose regulated protein 94)
**TRAP1**	TNF receptor associated protein 1	HSP75,<br>HSP90L[63]

Table S5. Nomenclature of chaperonin family members (Modified according to Kampinga et al[1] and HUGO Gene Nomenclature Committee). Citations elucidate the biological role of each family member.

Heat Shock Protein	Full Name	Aliases
**BBS10**	Bardet-Biedl syndrome 10	FLJ23560[64]
**BBS12[65]**	Bardet-Biedl syndrome 12	FLJ35630,<br>FLJ41559
**TCP1**	t-complex 1	D6S230E,<br>CCT1 (Chaperonin containing Tcp 1)[66],<br>Ccta
**CCT2[67]**	Chaperonin containing TCP1 subunit 2	Cctb
**CCT3[67]**	Chaperonin containing TCP1 subunit 3	Cctg
**CCT4[67]**	Chaperonin containing TCP1 subunit 4	Cctd
**CCT5[67]**	Chaperonin containing TCP1 subunit 5	KIAA0098
**CCT6A[68]**	Chaperonin containing TCP1 subunit 6A	TTCP20,<br>TCPZ,<br>Cctz,<br>HTR3 (Histidine transport regulator 3),<br>TCP20
**CCT6B**	Chaperonin containing TCP1 subunit 6B	Cctz2,<br>TSA303 (Testis specific protein 303)[69]
**CCT7**	Chaperonin containing TCP1 subunit 7	Ccth,<br>Nip7-1 [70]
**CCT8**	Chaperonin containing TCP1 subunit 8	Cctq,<br>PRED71
**CLPB**	caseinolytic mitochondrial matrix peptidase chaperone subunit B	HSP78,<br>SKD3[71],<br>FLJ13152,<br>ANKCLB(ankyrin repeat containing bacterial clp fusion)
**HSPD1**	heat shock protein family D (Hsp60) member 1	GroEL,<br>HSP60[72]
**HSPE1**	heat shock protein family E (Hsp10) member 1	CPN10[73],<br>GroES,<br>HSP10,<br>EPF
**MKKS[74]**	MKKS centrosomal shuttling protein	

Literature:

1. Kampinga, H.H.; Hageman, J.; Vos, M.J.; Kubota, H.; Tanguay, R.M.; Bruford, E.A.; Cheetham, M.E.; Chen, B.; Hightower, L.E. Guidelines for the nomenclature of the human heat shock proteins. *Cell Stress Chaperones* **2009**, *14*, 105–111, doi:10.1007/s12192-008-0068-7.

2. Ismailov, S.M.; Fedotov, V.P.; Dadali, E.L.; Polyakov, A.V.; Van Broeckhoven, C.; Ivanov, V.I.; De Jonghe, P.; Timmerman, V.; Evgrafov, O.V. A new locus for autosomal dominant Charcot-Marie-Tooth disease type 2 (CMT2F) maps to chromosome 7q11-q21. *Eur J. Hum. Genet.* **2001**, *9*, 646–650, doi:10.1038/sj.ejhg.5200686.
3. Suzuki, A.; Sugiyama, Y.; Hayashi, Y.; Nyu-i, N.; Yoshida, M.; Nonaka, I.; Ishiura, S.; Arahata, K.; Ohno, S. MKBP, a novel member of the small heat shock protein family, binds and activates the myotonic dystrophy protein kinase. *J. Cell Biol* **1998**, *140*, 1113–1124, doi:10.1083/jcb.140.5.1113.
4. Lam, W.Y.; Wing Tsui, S.K.; Law, P.T.; Luk, S.C.; Fung, K.P.; Lee, C.Y.; Waye, M.M. Isolation and characterization of a human heart cDNA encoding a new member of the small heat shock protein family--HSPL27. *Biochim Biophys Acta* **1996**, *1314*, 120–124, doi:10.1016/s0167-4889(96)00121-8.
5. Lee, S.; Carson, K.; Rice-Ficht, A.; Good, T. Hsp20, a novel alpha-crystallin, prevents Abeta fibril formation and toxicity. *Protein Sci* **2005**, *14*, 593–601, doi:10.1110/ps.041020705.
6. Charpentier, A.H.; Bednarek, A.K.; Daniel, R.L.; Hawkins, K.A.; Laflin, K.J.; Gaddis, S.; MacLeod, M.C.; Aldaz, C.M. Effects of estrogen on global gene expression: Identification of novel targets of estrogen action. *Cancer Res.* **2000**, *60*, 5977–5983.
7. Tang, B.S.; Zhao, G.H.; Luo, W.; Xia, K.; Cai, F.; Pan, Q.; Zhang, R.X.; Zhang, F.F.; Liu, X.M.; Chen, B.; et al. Small heat-shock protein 22 mutated in autosomal dominant Charcot-Marie-Tooth disease type 2L. *Hum. Genet.* **2005**, *116*, 222–224, doi:10.1007/s00439-004-1218-3.
8. Lechtreck, K.F.; Luro, S.; Awata, J.; Witman, G.B. HA-tagging of putative flagellar proteins in Chlamydomonas reinhardtii identifies a novel protein of intraflagellar transport complex B. *Cell Motil Cytoskeleton* **2009**, *66*, 469–482, doi:10.1002/cm.20369.
9. Davis, A.R.; Alevy, Y.G.; Chellaiah, A.; Quinn, M.T.; Mohanakumar, T. Characterization of HDJ-2, a human 40 kD heat shock protein. *Int J. Biochem Cell Biol* **1998**, *30*, 1203–1221, doi:10.1016/s1357-2725(98)00091-0.
10. Edwards, M.C.; Liegeois, N.; Horecka, J.; DePinho, R.A.; Sprague, G.F., Jr.; Tyers, M.; Elledge, S.J. Human CPR (cell cycle progression restoration) genes impart a Far- phenotype on yeast cells. *Genetics* **1997**, *147*, 1063–1076.
11. Linnoila, J.; Wang, Y.; Yao, Y.; Wang, Z.Z. A mammalian homolog of Drosophila tumorous imaginal discs, Tid1, mediates agrin signaling at the neuromuscular junction. *Neuron* **2008**, *60*, 625–641, doi:10.1016/j.neuron.2008.09.025.
12. Hoe, K.L.; Won, M.; Chung, K.S.; Jang, Y.J.; Lee, S.B.; Kim, D.U.; Lee, J.W.; Yun, J.H.; Yoo, H.S. Isolation of a new member of DnaJ-like heat shock protein 40 (Hsp40) from human liver. *Biochim Biophys Acta* **1998**, *1383*, 4–8, doi:10.1016/s0167-4838(97)00207-0.
13. Chen, M.S.; Roti, J.R.; Laszlo, A. Hsc40, a new member of the hsp40 family, exhibits similar expression profile to that of hsc70 in mammalian cells. *Gene* **1999**, *238*, 333–341, doi:10.1016/s0378-1119(99)00333-9.
14. Cornec-Le Gall, E.; Olson, R.J.; Besse, W.; Heyer, C.M.; Gainullin, V.G.; Smith, J.M.; Audrezet, M.P.; Hopp, K.; Porath, B.; Shi, B.; et al. Monoallelic Mutations to DNAJB11 Cause Atypical Autosomal-Dominant Polycystic Kidney Disease. *Am. J. Hum. Genet.* **2018**, *102*, 832–844, doi:10.1016/j.ajhg.2018.03.013.
15. El Khouri, E.; Thomas, L.; Jeanson, L.; Bequignon, E.; Vallette, B.; Duquesnoy, P.; Montantin, G.; Copin, B.; Dastot-Le Moal, F.; Blanchon, S.; et al. Mutations in DNAJB13, Encoding an HSP40 Family Member, Cause Primary Ciliary Dyskinesia and Male Infertility. *Am. J. Hum. Genet.* **2016**, *99*, 489–500, doi:10.1016/j.ajhg.2016.06.022.
16. Sopha, P.; Kadokura, H.; Yamamoto, Y.H.; Takeuchi, M.; Saito, M.; Tsuru, A.; Kohno, K. A novel mammalian ER-located J-protein, DNAJB14, can accelerate ERAD of misfolded membrane proteins. *Cell Struct Funct* **2012**, *37*, 177–187, doi:10.1247/csf.12017.
17. Brightman, S.E.; Blatch, G.L.; Zetter, B.R. Isolation of a mouse cDNA encoding MTJ1, a new murine member of the DnaJ family of proteins. *Gene* **1995**, *153*, 249–254, doi:10.1016/0378-1119(94)00741-a.
18. Hundley, H.A.; Walter, W.; Bairstow, S.; Craig, E.A. Human Mpp11 J protein: Ribosome-tethered molecular chaperones are ubiquitous. *Science* **2005**, *308*, 1032–1034, doi:10.1126/science.1109247.
19. Melville, M.W.; Katze, M.G.; Tan, S.L. P58IPK, a novel cochaperone containing tetratricopeptide repeats and a J-domain with oncogenic potential. *Cell Mol. Life Sci* **2000**, *57*, 311–322, doi:10.1007/PL00000692.
20. Silins, G.; Grimmond, S.; Hayward, N. Characterisation of a new human and murine member of the DnaJ family of proteins. *Biochem Biophys Res. Commun* **1998**, *243*, 273–276, doi:10.1006/bbrc.1997.8091.
21. Fontaine, S.N.; Zheng, D.; Sabbagh, J.J.; Martin, M.D.; Chaput, D.; Darling, A.; Trotter, J.H.; Stothert, A.R.; Nordhues, B.A.; Lussier, A.; et al. DnaJ/Hsc70 chaperone complexes control the extracellular release of neurodegenerative-associated proteins. *EMBO J.* **2016**, *35*, 1537–1549, doi:10.15252/embj.201593489.
22. Gundersen, C.B.; Kohan, S.A.; Souda, P.; Whitelegge, J.P.; Umbach, J.A. Cysteine string protein beta is prominently associated with nerve terminals and secretory organelles in mouse brain. *Brain Res.* **2010**, *1332*, 1–11, doi:10.1016/j.brainres.2010.03.044.
23. Edvardson, S.; Cinnamon, Y.; Ta-Shma, A.; Shaag, A.; Yim, Y.I.; Zenvirt, S.; Jalas, C.; Lesage, S.; Brice, A.; Taraboulos, A.; et al. A deleterious mutation in DNAJC6 encoding the neuronal-specific clathrin-uncoating co-chaperone auxilin, is associated with juvenile parkinsonism. *PLoS ONE* **2012**, *7*, e36458, doi:10.1371/journal.pone.0036458.
24. Cao, M.; Wu, Y.; Ashrafi, G.; McCartney, A.J.; Wheeler, H.; Bushong, E.A.; Boassa, D.; Ellisman, M.H.; Ryan, T.A.; De Camilli, P. Parkinson Sac Domain Mutation in Synaptojanin 1 Impairs Clathrin Uncoating at Synapses and Triggers Dystrophic Changes in Dopaminergic Axons. *Neuron* **2017**, *93*, 882–896 e885, doi:10.1016/j.neuron.2017.01.019.
25. Brychzy, A.; Rein, T.; Winklhofer, K.F.; Hartl, F.U.; Young, J.C.; Obermann, W.M. Cofactor Tpr2 combines two TPR domains and a J domain to regulate the Hsp70/Hsp90 chaperone system. *EMBO J.* **2003**, *22*, 3613–3623, doi:10.1093/emboj/cdg362.

26. Ito, N.; Kamiguchi, K.; Nakanishi, K.; Sokolovskya, A.; Hirohashi, Y.; Tamura, Y.; Murai, A.; Yamamoto, E.; Kanaseki, T.; Tsukahara, T.; et al. A novel nuclear DnaJ protein, DNAJC8, can suppress the formation of spinocerebellar ataxia 3 polyglutamine aggregation in a J-domain independent manner. *Biochem Biophys Res. Commun* **2016**, *474*, 626–633, doi:10.1016/j.bbrc.2016.03.152.
27. Han, C.; Chen, T.; Li, N.; Yang, M.; Wan, T.; Cao, X. HDJC9, a novel human type C DnaJ/HSP40 member interacts with and cochaperones HSP70 through the J domain. *Biochem Biophys Res. Commun* **2007**, *353*, 280–285, doi:10.1016/j.bbrc.2006.12.013.
28. Takamura, Y.; Yagi, H.; Hase, K.; Yoneda, T.; Maeda, M.; Akagi, Y.; Sato, M. JDD1, a novel member of the DnaJ family, is expressed in the germinal zone of the rat brain. *Biochem Biophys Res. Commun* **2001**, *285*, 387–392, doi:10.1006/bbrc.2001.5181.
29. Cunnea, P.M.; Miranda-Vizuete, A.; Bertoli, G.; Simmen, T.; Damdimopoulos, A.E.; Hermann, S.; Leinonen, S.; Huikko, M.P.; Gustafsson, J.A.; Sitia, R.; et al. ERdj5, an endoplasmic reticulum (ER)-resident protein containing DnaJ and thioredoxin domains, is expressed in secretory cells or following ER stress. *J. Biol Chem* **2003**, *278*, 1059–1066, doi:10.1074/jbc.M206995200.
30. Violitzi, F.; Perivolidi, V.I.; Thireou, T.; Grivas, I.; Haralambous, S.; Samiotaki, M.; Panayotou, G.; Douni, E. Mapping Interactome Networks of DNAJC11, a Novel Mitochondrial Protein Causing Neuromuscular Pathology in Mice. *J. Proteome Res.* **2019**, *18*, 3896–3912, doi:10.1021/acs.jproteome.9b00338.
31. Choi, J.; Djebbar, S.; Fournier, A.; Labrie, C. The co-chaperone DNAJC12 binds to Hsc70 and is upregulated by endoplasmic reticulum stress. *Cell Stress Chaperones* **2014**, *19*, 439–446, doi:10.1007/s12192-013-0471-6.
32. Bermak, J.C.; Li, M.; Bullock, C.; Zhou, Q.Y. Regulation of transport of the dopamine D1 receptor by a new membrane-associated ER protein. *Nat. Cell Biol* **2001**, *3*, 492–498, doi:10.1038/35074561.
33. Navasa, N.; Martin-Ruiz, I.; Atondo, E.; Sutherland, J.D.; Angel Pascual-Itoiz, M.; Carreras-Gonzalez, A.; Izadi, H.; Tomas-Cortazar, J.; Ayaz, F.; Martin-Martin, N.; et al. Ikaros mediates the DNA methylation-independent silencing of MCJ/DNAJC15 gene expression in macrophages. *Sci Rep.* **2015**, *5*, 14692, doi:10.1038/srep14692.
34. Yamamoto, Y.H.; Noda, T. Autophagosome formation in relation to the endoplasmic reticulum. *J. Biomed. Sci* **2020**, *27*, 97, doi:10.1186/s12929-020-00691-6.
35. Pascarella, A.; Ferrandino, G.; Credendino, S.C.; Moccia, C.; D'Angelo, F.; Miranda, B.; D'Ambrosio, C.; Bielli, P.; Spadaro, O.; Ceccarelli, M.; et al. DNAJC17 is localized in nuclear speckles and interacts with splicing machinery components. *Sci Rep.* **2018**, *8*, 7794, doi:10.1038/s41598-018-26093-1.
36. Gomes, C.; Soh, J. DnaJC18, a Novel Type III DnaJ Family Protein, is Expressed Specifically in Rat Male Germ Cells. *Dev. Reprod* **2017**, *21*, 237–247, doi:10.12717/DR.2017.21.3.237.
37. Heinemeyer, T.; Stemmet, M.; Bardien, S.; Neethling, A. Underappreciated Roles of the Translocase of the Outer and Inner Mitochondrial Membrane Protein Complexes in Human Disease. *DNA Cell Biol* **2019**, *38*, 23–40, doi:10.1089/dna.2018.4292.
38. Vickery, L.E.; Silberg, J.J.; Ta, D.T. Hsc66 and Hsc20, a new heat shock cognate molecular chaperone system from Escherichia coli. *Protein Sci* **1997**, *6*, 1047–1056, doi:10.1002/pro.5560060511.
39. Warren, A.J. Molecular basis of the human ribosomopathy Shwachman-Diamond syndrome. *Adv. Biol Regul* **2018**, *67*, 109–127, doi:10.1016/j.jbior.2017.09.002.
40. Wingen, C.; Aschenbrenner, A.C.; Stumpges, B.; Hoch, M.; Behr, M. The Wurst protein: A novel endocytosis regulator involved in airway clearance and respiratory tube size control. *Cell Adh Migr* **2009**, *3*, 14–18, doi:10.4161/cam.3.1.7088.
41. Roosen, D.A.; Blauwendraat, C.; Cookson, M.R.; Lewis, P.A. DNAJC proteins and pathways to parkinsonism. *FEBS J.* **2019**, *286*, 3080–3094, doi:10.1111/febs.14936.
42. Nepomuceno-Silva, J.L.; de Melo, L.D.; Mendonca, S.M.; Paixao, J.C.; Lopes, U.G. RJLs: A new family of Ras-related GTP-binding proteins. *Gene* **2004**, *327*, 221–232, doi:10.1016/j.gene.2003.11.010.
43. Anderson, J.F.; Siller, E.; Barral, J.M. The neurodegenerative-disease-related protein sacsin is a molecular chaperone. *J. Mol. Biol* **2011**, *411*, 870–880, doi:10.1016/j.jmb.2011.06.016.
44. Wiggs, J.L. DNAJC30 biallelic mutations extend mitochondrial complex I-deficient phenotypes to include recessive Leber's hereditary optic neuropathy. *J. Clin. Invest.* **2021**, *131*, doi:10.1172/JCI147734.
45. Hunt, C.R.; Gasser, D.L.; Chaplin, D.D.; Pierce, J.C.; Kozak, C.A. Chromosomal localization of five murine HSP70 gene family members: Hsp70-1, Hsp70-2, Hsp70-3, Hsc70t, and Grp78. *Genomics* **1993**, *16*, 193–198, doi:10.1006/geno.1993.1158.
46. Milner, C.M.; Campbell, R.D. Structure and expression of the three MHC-linked HSP70 genes. *Immunogenetics* **1990**, *32*, 242–251, doi:10.1007/BF00187095.
47. Dressel, R.; Gunther, E. Heat-induced expression of MHC-linked HSP70 genes in lymphocytes varies at the single-cell level. *J. Cell Biochem* **1999**, *72*, 558–569.
48. Schulte, A.M.; Fischer, S.; Sachse, G.E.; Hafner, S.; Stelck, S.; Gassen, H.G. Identification and characterization of a novel hsc70-like gene in the human lung tumor cell line HS24. *DNA Cell Biol* **1997**, *16*, 257–268, doi:10.1089/dna.1997.16.257.
49. Nonoguchi, K.; Itoh, K.; Xue, J.H.; Tokuchi, H.; Nishiyama, H.; Kaneko, Y.; Tatsumi, K.; Okuno, H.; Tomiwa, K.; Fujita, J. Cloning of human cDNAs for Apg-1 and Apg-2, members of the Hsp110 family, and chromosomal assignment of their genes. *Gene* **1999**, *237*, 21–28, doi:10.1016/s0378-1119(99)00325-x.
50. Pobre, K.F.R.; Poet, G.J.; Hendershot, L.M. The endoplasmic reticulum (ER) chaperone BiP is a master regulator of ER functions: Getting by with a little help from ERdj friends. *J. Biol Chem* **2019**, *294*, 2098–2108, doi:10.1074/jbc.REV118.002804.
51. Noonan, E.J.; Place, R.F.; Giardina, C.; Hightower, L.E. Hsp70B' regulation and function. *Cell Stress Chaperones* **2007**, *12*, 219–229, doi:10.1379/csc-278.1.

52. Parsian, A.J.; Sheren, J.E.; Tao, T.Y.; Goswami, P.C.; Malyapa, R.; Van Rheeden, R.; Watson, M.S.; Hunt, C.R. The human Hsp70B gene at the HSPA7 locus of chromosome 1 is transcribed but non-functional. *Biochim Biophys Acta* **2000**, *1494*, 201–205, doi:10.1016/s0167-4781(00)00203-7.
53. Bruce, B.D.; Churchich, J. Characterization of the molecular-chaperone function of the heat-shock-cognate-70-interacting protein. *Eur J. Biochem* **1997**, *245*, 738–744, doi:10.1111/j.1432-1033.1997.00738.x.
54. Wadhwa, R.; Taira, K.; Kaul, S.C. An Hsp70 family chaperone, mortalin/mthsp70/PBP74/Grp75: What, when, and where? *Cell Stress Chaperones* **2002**, *7*, 309–316, doi:10.1379/1466-1268(2002)007<0309:ahfcmm>2.0.co;2.
55. Brocchieri, L.; Conway de Macario, E.; Macario, A.J. hsp70 genes in the human genome: Conservation and differentiation patterns predict a wide array of overlapping and specialized functions. *BMC Evol Biol* **2008**, *8*, 19, doi:10.1186/1471-2148-8-19.
56. He, Y.; Xu, R.; Zhai, B.; Fang, Y.; Hou, C.; Xing, C.; Xiao, H.; Chen, G.; Wang, X.; Ma, N.; et al. Hspa13 Promotes Plasma Cell Production and Antibody Secretion. *Front. Immunol* **2020**, *11*, 913, doi:10.3389/fimmu.2020.00913.
57. Wan, T.; Zhou, X.; Chen, G.; An, H.; Chen, T.; Zhang, W.; Liu, S.; Jiang, Y.; Yang, F.; Wu, Y.; et al. Novel heat shock protein Hsp70L1 activates dendritic cells and acts as a Th1 polarizing adjuvant. *Blood* **2004**, *103*, 1747–1754, doi:10.1182/blood-2003-08-2828.
58. Hatayama, T.; Yasuda, K.; Nishiyama, E. Characterization of high-molecular-mass heat shock proteins and 42 degrees C-specific heat shock proteins of murine cells. *Biochem Biophys Res. Commun* **1994**, *204*, 357–365, doi:10.1006/bbrc.1994.2467.
59. Kusaczuk, M.; Cechowska-Pasko, M. Molecular chaperone ORP150 in ER stress-related diseases. *Curr Pharm Des.* **2013**, *19*, 2807–2818, doi:10.2174/1381612811319150016.
60. Wang, H.; Pezeshki, A.M.; Yu, X.; Guo, C.; Subjeck, J.R.; Wang, X.Y. The Endoplasmic Reticulum Chaperone GRP170: From Immunobiology to Cancer Therapeutics. *Front. Oncol* **2014**, *4*, 377, doi:10.3389/fonc.2014.00377.
61. Haase, M.; Fitze, G. HSP90AB1: Helping the good and the bad. *Gene* **2016**, *575*, 171–186, doi:10.1016/j.gene.2015.08.063.
62. Binder, R.J.; Han, D.K.; Srivastava, P.K. CD91: A receptor for heat shock protein gp96. *Nat. Immunol* **2000**, *1*, 151–155, doi:10.1038/77835.
63. Felts, S.J.; Owen, B.A.; Nguyen, P.; Trepel, J.; Donner, D.B.; Toft, D.O. The hsp90-related protein TRAP1 is a mitochondrial protein with distinct functional properties. *J. Biol Chem* **2000**, *275*, 3305–3312, doi:10.1074/jbc.275.5.3305.
64. Stoetzel, C.; Laurier, V.; Davis, E.E.; Muller, J.; Rix, S.; Badano, J.L.; Leitch, C.C.; Salem, N.; Chouery, E.; Corbani, S.; et al. BBS10 encodes a vertebrate-specific chaperonin-like protein and is a major BBS locus. *Nat. Genet.* **2006**, *38*, 521–524, doi:10.1038/ng1771.
65. Alvarez-Satta, M.; Castro-Sanchez, S.; Valverde, D. Bardet-Biedl Syndrome as a Chaperonopathy: Dissecting the Major Role of Chaperonin-Like BBS Proteins (BBS6-BBS10-BBS12). *Front. Mol. Biosci* **2017**, *4*, 55, doi:10.3389/fmolb.2017.00055.
66. Wang, D.Y.; Kamuda, K.; Montoya, G.; Mesa, P. The TRiC/CCT Chaperonin and Its Role in Uncontrolled Proliferation. *Adv. Exp. Med. Biol* **2020**, *1243*, 21–40, doi:10.1007/978-3-030-40204-4_2.
67. Horwich, A.L.; Fenton, W.A. Chaperonin-assisted protein folding: A chronologue. *Q Rev. Biophys* **2020**, *53*, e4, doi:10.1017/S0033583519000143.
68. Wei, Q.; Zhu, G.; Cui, X.; Kang, L.; Cao, D.; Jiang, Y. Expression of CCT6A mRNA in chicken granulosa cells is regulated by progesterone. *Gen. Comp. Endocrinol* **2013**, *189*, 15–23, doi:10.1016/j.ygcen.2013.04.019.
69. Ozaki, K.; Kuroki, T.; Hayashi, S.; Nakamura, Y. Isolation of three testis-specific genes (TSA303, TSA806, TSA903) by a differential mRNA display method. *Genomics* **1996**, *36*, 316–319, doi:10.1006/geno.1996.0467.
70. Zanchin, N.I.; Roberts, P.; DeSilva, A.; Sherman, F.; Goldfarb, D.S. Saccharomyces cerevisiae Nip7p is required for efficient 60S ribosome subunit biogenesis. *Mol. Cell Biol* **1997**, *17*, 5001–5015, doi:10.1128/MCB.17.9.5001.
71. Perier, F.; Radeke, C.M.; Raab-Graham, K.F.; Vandenberg, C.A. Expression of a putative ATPase suppresses the growth defect of a yeast potassium transport mutant: Identification of a mammalian member of the Clp/HSP104 family. *Gene* **1995**, *152*, 157–163, doi:10.1016/0378-1119(94)00697-q.
72. Venner, T.J.; Singh, B.; Gupta, R.S. Nucleotide sequences and novel structural features of human and Chinese hamster hsp60 (chaperonin) gene families. *DNA Cell Biol* **1990**, *9*, 545–552, doi:10.1089/dna.1990.9.545.
73. Georgopoulos, C.; Welch, W.J. Role of the major heat shock proteins as molecular chaperones. *Annu Rev. Cell Biol* **1993**, *9*, 601–634, doi:10.1146/annurev.cb.09.110193.003125.
74. Scott, C.A.; Marsden, A.N.; Rebagliati, M.R.; Zhang, Q.; Chamling, X.; Searby, C.C.; Baye, L.M.; Sheffield, V.C.; Slusarski, D.C. Nuclear/cytoplasmic transport defects in BBS6 underlie congenital heart disease through perturbation of a chromatin remodeling protein. *PLoS Genet.* **2017**, *13*, e1006936, doi:10.1371/journal.pgen.1006936.
